# Vaginal Dysplasia and HIV: An African American and Caribbean American Cohort Study

**DOI:** 10.1155/2019/6189837

**Published:** 2019-01-01

**Authors:** Lunthita M. Duthely, Jose A. Carugno, Cayla Y. Suthumphong, Erica B. Feldman, JoNell E. Potter

**Affiliations:** ^1^Department of Obstetrics and Gynecology, Division of Research and Special Projects Miami, University of Miami Miller School of Medicine, Miami 33101, USA; ^2^Department of Obstetrics and Gynecology, University of Miami Miller School of Medicine, Miami 33101, USA; ^3^Department of Medical Education, University of Miami Miller School of Medicine, Miami 33101, USA

## Abstract

**Background:**

Vaginal cancer is a rare disease with poor clinical outcomes and limited therapeutic options. In the United States (US), minority women and older women are disproportionately diagnosed with late-stage vaginal cancer. Sociodemographic characteristics, risk behaviors, and cooccurring conditions are linked to vaginal intraepithelial neoplasia (VaIN). The diagnosis of VaIN is more prevalent among older women and women living with HIV (WLWH). The Caribbean basin has one of the highest rates of anogenital cancers in the Western Hemisphere. In the US, vaginal infections are more prevalent among Caribbean women, and these infections contribute to higher rates of Human Papilloma Virus (HPV). Given the high rate of anogenital cancers in the Caribbean and the high rates of HPV among Caribbean women in the US, we sought to describe the occurrence of VaIN in a cohort of Black non-Hispanic WLWH. The cohort was followed by an interdisciplinary team of providers with the University of Miami, Miller School of Medicine.

**Results:**

Caribbean Americans were living with HIV longer and more were uninsured; more African Americans endorsed cigarette and illicit substance use. Caribbean Americans trended towards the highest grades of VaIN (VaIN 2+) at baseline, but more African Americans progressed to VaIN 2+ in subsequent biopsies.

**Conclusion:**

In this cohort of Caribbean American and African American women living with HIV diagnosed with VaIN, Caribbean Americans had the highest grade of VaIN at baseline, but more African Americans progressed to more advanced stages of the disease.

## 1. Introduction

Vaginal cancer is a rare disease that is difficult to treat, often with poor clinical outcomes [1]. More importantly, therapeutic options for vaginal cancer are limited [2], and certain treatment options, such as a vaginectomy, worsen the individual's quality of life [3]. Smoking, early sexual debut, multiple sexual partners, diagnosis of other anogenital cancers, and a prior hysterectomy are risk factors associated with vaginal cancer [4, 5]. In the United States (US), disparities by demographic characteristics have been demonstrated as well. Black non-Hispanic and Hispanic women are more likely to be diagnosed with late-stage (invasive) vaginal cancer—as are older women across all racial/ethnic groups US [5].

The precursor to vaginal cancer is vaginal intraepithelial neoplasia (VaIN), a Human Papilloma Virus- (HPV-) related premalignant condition [6]. VaIN is an important disease to understand, because of the associated risk of progression to vaginal cancer [7]. Studies examining VaIN are limited, however, due to the rarity of its occurrence in the general population [8]; however, previous epidemiological studies (15 to 40 years earlier) estimated the incidence of VaIN to be approximately .2 to .3 per 100,000 for women living in the US [9, 10] [11] and in Europe [12–14]. There is, unfortunately, a high rate of relapse of VaIN, which is associated with HPV positivity and the presence of HPV condylomas (anogenital warts) [15]. The diagnosis of VaIN is more prevalent among women who are immunosuppressed, including women living with HIV [16].

The epidemiology of VaIN among women living with HIV (WLWH) in the US is even less known, with a paucity of studies examining the cooccurrence of VaIN and HIV. A recent, 12-year, multicenter, study reported on the vaginal biopsies of 255 WLWH (67% Black, 18% Hispanic), where 53% were diagnosed with VaIN 1, 15% were with VaIN 2 or higher, and 33% of the biopsies were negative for VaIN [17]. Smeltzer et al. [18] reported on 54 posthysterectomy WLWH (80% Black, 12% Hispanic): 43% were diagnosed VaIN 1, 29% were VaIN 2 or higher, and 28% were negative for VaIN [18]. Risk factors, including smoking, low CD4 counts, high HIV viral load counts, and not taking antiretroviral medication (ARV) were associated with VaIN diagnosis [17]. Posthysterectomy WLWH are more likely to be diagnosed with abnormal Pap smear results and VaIN, compared to posthysterectomy HIV-seronegative women [17, 18].

Miami-Dade County (Miami) has the highest HIV transmission rate in the US, and non-Hispanic Blacks are disproportionately affected by the HIV epidemic [19]. In fact, when the demographic profile of Miami is disaggregated by ethnic groups, Caribbeans—including US-born and foreign-born individuals from Haiti, Jamaica, and other Caribbean nations—make up 33% of the Black non-Hispanic population of Miami [19]. Most US-based studies aggregate Black non-Hispanics into one racial/ethnic group when describing VaIN and vaginal cancer. In this study, the data is disaggregated across two major ethnic groups of non-Hispanic women of Black race—African American and Caribbean American.

In Miami, this distinction is important for several reasons: (1) the highest rate of anogenital cancers in the Western Hemisphere occur among women living in Caribbean [20]; and (2) Haitian women living in Miami—US-born and foreign-born [21] and Haitian women living in Haiti [22, 23]—are more likely to be diagnosed with HPV and other vaginal infections linked to anogenital cancers [23]. Comparing the demographic and clinical profile of non-Hispanic WLHW of the Black race diagnosed with VaIN may offer insight into the epidemiology of this rare condition among non-Hispanic Blacks, who bear a disproportionate burden of VaIN, vaginal cancer, and HIV. The purpose of this study is to compare the demographic characteristics and initial clinical outcomes of non-Hispanic Black WLHW diagnosed with VaIN in the largest, specialty care gynecology HIV clinic dedicated to anogenital dysplasia screening in Miami. The clinic is managed by an interdisciplinary team of Gynecological Oncology and Infectious Disease specialists.

## 2. Study Methods

### 2.1. Study Design

This was a retrospective cohort study using an electronic database that captures gynecological care of women living with HIV. Approximately 1,600 unique women of Black race were captured in this database. A convenience sample of vaginal biopsy data collected from January 1, 2009, to December 31, 2016, was extracted. Records with complete information on HIV diagnosis date, racial/ethnic background, age, biopsy result, and documented antiretroviral history were included. Socioeconomic status, risk behaviors, and associated cervical Pap smear result at baseline were extracted for patients who underwent one or more a vaginal biopsies. The records of 63 African American and Caribbean American women biopsied for suspicious vaginal lesions met criteria. The study was approved by the University of Miami Institutional Review Board for human subjects research.

### 2.2. Statistical Analyses

The data were stratified by ethnic group. The Chi-Square statistic was used to compare categorical variables by the two ethnic groups. Fisher's exact test was executed in place of the Chi-Square for tables with small frequency cells (< 5). Student's t-test compared continuous variables for the two ethnic groups; means and standard deviations (SD) were reported. The data were analyzed with SPSS 22 [24].

## 3. Results

### 3.1. Baseline Characteristics

This study included 63 patients who met criteria. Baseline demographic, socioeconomic status, and social-behavioral and clinical characteristics were analyzed by ethnic group (see Table 1). Caribbean Americans were older by three years (55.5±7.3 vs. 52.3±7.6) and had been living with HIV approximately three years longer than the African Americans (14.4 ± 7.2 vs. 11.3 ± 6.9 years). A higher proportion (79% vs. 59%) of Caribbean Americans were over the age of 50; the difference by age group approached (p=0.05) but did not reach significance. Whereas a significantly higher proportion of African Americans, compared to Caribbean Americans (77% vs. 46%; p=0.011), relied on Medicaid/Medicare as their primary source of healthcare reimbursement, a significantly higher proportion of Caribbean Americans were uninsured (46% vs. 15%; p=0.006). African Americans were more likely to report past or current history of cigarette use (56% vs. 8%; p≤0.001). No (0%) Caribbean Americans, compared to 33% of African Americans, endorsed past or current use of illicit substances (p≤0.001). The majority of patients across both ethnic groups were virologically suppressed (<200 copies/mL), prescribed ARV, and not severely immunocompromised (i.e., CD4≥200) at baseline; no significant differences were found overall by viral load suppression or ARV use. (see Table 1).

### 3.2. Gynecological History at Baseline

Baseline gynecological history and vaginal biopsies are summarized in Table 2. An overwhelming majority of patients among both the African American cohort and the Caribbean American cohort, 90% and 88%, respectively, were posthysterectomy. For a higher proportion of Caribbean Americans, fibroids and dysplasia, 45% and 30%, respectively, were the primary indications for the hysterectomy. There was a trend towards higher grades of abnormalities on baseline Pap smear results for the African American cohort. Low Grade SIL (LSIL) was found for 61% of the African Americans and 50% of Caribbean Americans. One high grade/invasive cancer Pap result occurred among the African American group (see Table 2).

Table 3 summarizes the vaginal biopsy results at baseline and follow-up, stratified by ethnic group. For more meaningful comparisons, biopsy results were reduced to three categories: negative/benign, VaIN 1 or VaIN 2 or higher (VaIN 2+). A significantly higher proportion of African Americans were VaIN 1 on baseline vaginal biopsy, compared to Caribbean Americans (64% vs. 33%, p=0.017). A higher proportion of Caribbean Americans were either negative at baseline vaginal biopsy (42% vs. 23%, p=0.11) or were VaIN 2 or higher (25% vs. 13%; p=0.21), but the differences were not significant. Among this cohort of women biopsied for suspicious vaginal lesions, 13% of the African Americans and 25% of Caribbean Americans were diagnosed with VaIN 2+ at baseline (Table 3). A total of 11 women, out of approximately 1,600 non-Hispanic Black WLWH receiving gynecological care in the clinic, were diagnosed with VaIN 2+—yielding a rate of approximately 6.9 per 1000.

The follow-up biopsy data were then reduced to two categories to indicate progression or nonprogression (Table 3). The “nonprogression/regressed” category included the subsequent biopsies that regressed to a lower grade VaIN or remained at the same grade. Although the differences were not significant, a larger proportion of African Americans progressed to higher grades of VaIN (58% vs. 25%; p=0.13), compared to Caribbean Americans.

### 3.3. Baseline by VaIN Grade by Ethnicity

We then further stratified patient baseline characteristics by VaIN grade within ethnic group, which is tabulated in Table 4. Among the Caribbean Americans, the use of ARVs was lower for those diagnosed with VaIN 2+ (83%), compared to those diagnosed with VaIN 1 (100%); also, a higher proportion of Caribbean Americans with VaIN 1 had CDC-defined AIDS (88%), compared to African Americans (80%).

The number of observations was small, but a few findings at baseline are worth noting (Table 4). We found 100% of patients with VaIN 2+ for both ethnic groups had CDC-defined AIDS. Across the four groups (VaIN grade within ethnic group), the following was observed: the highest proportion of detectable viral load occurred among the VaIN 1 (50%) of Caribbean Americans; the lowest rate of ARV treatment occurred among the VaIN 2+ of Caribbean Americans; the lowest rate of history of hysterectomy occurred among the VaIN 2+ of Caribbean Americans; yet, the highest rate of hysterectomy due to dysplasia occurred among the VaIN 2+ of Caribbean Americans, and the highest rate of hysterectomy due to fibroids occurred among the Caribbean Americans.

When examined by baseline vaginal biopsy, we found the following with respect to cigarette and illicit drug use. Among the African Americans, there was an equal proportion that endorsed cigarette use (VaIN 1 vs. VaIN 2+). The only smoker among the Caribbeans was found among one of the six patients diagnosed with VaIN 2+. No illicit substance use occurred among the Caribbean Americans; among the African Americans, 80% of VaIN 2+ and 40% of VaIN 1 endorsed illicit drug use (see Table 4).

## 4. Discussion

In this retrospective study of two ethnically diverse, major groups of non-Hispanic Black WLWH in the US, biopsied for vaginal lesions, 58% of the African Americans and 25% of the Caribbean Americans progressed to a higher grade VaIN at follow-up. Our baseline rate of 17% VaIN 2+ was slightly higher than the results of a recent, US-based, 12-year, multisite study of 255 WLWH (67% Black, 18% Hispanic)—where 15% were diagnosed with VaIN 2+ [17]—but lower than a cohort study reporting on 54 posthysterectomy WLWH (80% Black, 12% Hispanic), with a rate of 29% diagnosed with VaIN 2+ [18]. In our cohort, 11 out of approximately 1,600 women of Black race, receiving gynecological care from 2009-2016, were diagnosed with VaIN 2+—yielding an overall prevalence rate of approximately 6.9 per 1000 WLWH of Black race, diagnosed with VaIN 2+. To our knowledge, this represents one of the largest cohorts of WLWH, where patients were managed at a single institution, by an interdisciplinary team of Gynecological Oncology and Infectious Disease specialists. The diagnosis, care, and treatment of vaginal dysplasia were more or less homogeneous.

At baseline, where 89% of patients presented with a history of hysterectomy, there was a trend towards higher grades of abnormalities detected on Pap smear test for the African American group, compared to Caribbean Americans. It is known that posthysterectomy WLWH are more likely to be diagnosed with abnormal Pap test [17, 18]. The only case of high grade/invasive cancer detected on Pap test occurred among the African American group, who had the highest rate of cigarette and illicit substance use. Previous studies reported that smoking, not taking ARV medications, and high HIV viral load counts were associated with a VaIN diagnosis [17]. In this cohort, African Americans were significantly more likely to report past or current history of cigarette use (56% vs. 8%; p≤0.001). When stratified by VaIN grade within ethnic group, 60% of African Americans who were VaIN 2+ endorsed cigarette use. The only smoker among the Caribbeans was one of the six patients diagnosed with VaIN 2+. Among the African Americans, 80% of those diagnosed with VaIN 2+ endorsed illicit drug use. To date, no other study reported on illicit substance use and the occurrence of VaIN among WLWH. Consistent with other findings, smoking [17] (among the African-Americans in our cohort) was associated with a higher grade of VaIN in this cohort.

The lowest rate of ARV treatment and the highest rate of hysterectomy due to dysplasia occurred among the VaIN 2+ group of Caribbean Americans. Also, the highest rate of hysterectomy due to fibroids occurred among the Caribbean Americans. Consistent with other studies [17, 18], women who are posthysterectomy (majority of the cohort) are more likely to be diagnosed with VaIN, and not taking ARV (among the Caribbean-Americans in our cohort) is associated with higher grade VaIN.

Length of time living in the US among the Caribbean Americans was not captured in the database; and so it is possible that a proportion of the Caribbeans had been living in the US for a shorter time, had not established residency in the state, and were therefore uninsured and not taking ARVs. It is possible that these women sought care later in the disease process at the time of hysterectomy—hence the higher rate of a history of hysterectomy due to dysplasia—or when biopsied for suspicious vaginal lesions—hence the higher rate of VaIN 2+ at baseline. It has been shown in the general population that shorter time in the US for immigrants is associated with a lower prevalence of cigarette and illicit drug use, which could account for the significantly lower proportion of cigarette use (8% vs. 56%) and illicit drug use (0% vs. 33%) for the Caribbean Americans, compared to the African Americans.

It was not possible to fully assess HPV positivity, nor the true rate of VaIN recurrence in this cohort, but two interesting findings in the literature regarding HPV and VaIN recurrence are worth noting here. First, while HPV positivity is a known precursor to anogenital dysplasia and cancer, a recent meta-analysis demonstrated that HPV positivity in vulvar cancer and other cancers, head and neck cancer, for example, predicted significantly* longer* survival [25]. Second, studies that reported on factors associated with VaIN and its recurrence posttreatment suggested that among the general, non-HIV population the correlation may be due to closer monitoring that occurs after hysterectomy [15].

We acknowledge several limitations of our study. First, this was a retrospective analysis of information captured in a database designated for HIV general and specialty gynecological care. The dataset included data of WLWH biopsied for suspicious vaginal lesions. Second, the cohort consisted of a convenience sample of 63 WLWH biopsied from 2009 to 2016—historical information was not available for this analysis. Third, the results summarized for the follow-up biopsies are biased, as they are limited to data available in the database only. Information regarding loss-to-follow-up could not be ascertained. Fourth, the analyses were limited to variables coded in the database. Length of time in the US for the Caribbean Americans, for example, was not available but may have provided a better description about these women. Finally, screening for the oncogenic strains of HPV was not routinely conducted during the study period, which limited the ability to further describe VaIN abnormalities among this cohort.

This study is significant and relevant for several reasons. First, this study highlights the importance of understanding VaIN among two of the subgroups most disproportionally affected by VaIN—namely, older women and minorities. To our knowledge, this is the only published study disaggregating the occurrence of VaIN, a precursor to vaginal cancer, among US women of the Black race, in general, and specifically among WLWH. The analysis included a cohort of older, WLWH, self-identified as African American or Caribbean American. Vaginal cancer is largely preventable via the use of HPV vaccines, which target the most oncogenic HPV genotypes that contribute to the majority of vaginal cancers [26]; but vaccination is an option only for adolescents and young adults; furthermore, long-term survival, after vaccination, has yet to be determined [27]. Second, the occurrence of vaginal cancer is rare and treatment options are limited; yet, increasingly a smaller number of clinical trials are available to address the diagnoses of VaIN and vaginal cancer [28]. Third, the Caribbean American WLWH in Miami are predominantly of Haitian, Bahamian, and Jamaican ancestry. Certain personal hygiene practices documented among Haitian women living in Miami [21] and Haitian women living in Haiti [22, 23] were found to increase the susceptibility of contracting HPV [23]. These practices are thought to disturb the vaginal microbiome, which is associated with an increased risk of vaginal infections such as HPV [23]. Taken together, although descriptive in nature, this study is significant in that it addresses several gaps in the literature regarding the diagnosis of VaIN among older, minority women living with HIV in the US—specifically, women of the Black race.

Finally, the cooccurrence of VaIN and HIV is relevant particularly in regions with high HIV transmission rates, given the shared risk factors of the two conditions. Sociodemographic disparities, including racial/ethnic minority status and lower socioeconomic status, and behavioral risk factors—such as early sexual debut, multiple sexual partners, and cooccurrence of sexually transmitted infections—are risk factors shared by women living with HIV and women diagnosed with VaIN. Also, this is the only study that described the cohort by primary insurance coverage, a surrogate for socioeconomic status, and illicit substance use as well. We found significant differences among this cohort by insurance coverage and illicit substance use, where the Haitian Americans, who presented with higher grade VaIN, were not taking ARV and were uninsured at baseline, and the African Americans, who were more likely to endorse illicit substance use (and cigarette use), progressed to higher grade VaIN on follow-up. Furthermore, in the advent of highly active antiretroviral regimens, HIV can be managed as a chronic disease, and so individuals are living longer. Comprehensive, multisite, short-term, and longitudinal studies are needed to gain a better understanding of VaIN and vaginal cancer among older, minority women living with HIV in the United States.

## 5. Conclusions

In this cohort of Caribbean American and African American women living with HIV who were biopsied for vaginal lesions, Caribbean Americans had the highest grade of VaIN at initial diagnosis, but African Americans progressed to more advanced stages of the disease. Vaccination against the most oncogenic strains of HPV is a preventative measure that can be taken to reduce the risk of developing VaIN and vaginal cancer [26] but is not an option for older women. We, therefore, recommend a thorough evaluation of risk factors, targeted patient education, and closer follow-up of minority women diagnosed with VaIN to prevent potential progression of the disease.

## Figures and Tables

**Table 1:** Demographic and baseline characteristics by ethnicity (n=63).

	**African-American *(n=39)***	**Caribbean-American *(n=24)***	**Statistic**
**Age at VaIN Diagnosis** (years)			
** Mean (SD)**	52.3 (7.6)	55.5 (7.3)	p=0.10
** Range (min-max)**	34-70	39-71	
**Age Group**			p=0.15
< 50	13 (33)	4 (17)	p=0.05
50-64	23 (59)	19 (79)	
>=65	3 (8)	1 (4)	
**Socio-economic Status (SES)**			
Living at / below Poverty	26 (67)	18 (75)	p=0.58
**Insurance**			
Medicaid/Medicare	30 (77)	11 (46)	p=0.011
Uninsured	6 (15)	11 (46)	p=0.006
**Social History**			
Cigarette Use	22 (56)	2 (8)	p≤0.001
Illicit Drug Use	13 (33)	0 (0)	p≤0.001
**Length of Time HIV Diagnosed** (years)			
Range (years)	0-22	2-34	
Mean (SD)	11.3 (6.1)	14.4 (7.2)	p=0.13
**CDC-Defined AIDS**	33 (85)	22 (92)	p=0.69
**Viral Load Suppression **	***(n=36)***	***(n=23)***	
(<200 copies/mL)	30 (75)	17 (70)	p=0.38
**CD4**≥**200**	***(n=38)***	***(n=24)***	
(cells/*μ*L)	33 (87)	21 (88)	p=1
**On Anti-retrovirals (ARV)**	38 (97)	23 (96)	p=1

**Table 2:** Gynecological history at baseline, stratified by ethnic group.

	**African-American *(n=39)***	**Caribbean-American *(n=24)***	**Statistic**
**Hysterectomy**			
*∗*At baseline	35 (90)	21 (88)	p=1
**Hysterectomy Indication**		***(n=20)***	
Fibroids	13 (39)	9 (45)	p= 0.68
Dysplasia/Cancer	7 (21)	6 (30)	p= 0.47
Abnormal Bleeding	5 (15)	2 (10)	p= 0.59
Other Indications	8 (24)	3 (15)	p= 0.42
Unknown	2 ( - )	1 ( - )	-
**Pap Smear Result (Baseline)**	***(n=39)***	***(n=20)***	
Negative/ASCUS	14 (36)	10 (50)	p= 0.29
LGSIL	24 (61)	10 (50)	p=0.39
HGSIL/Invasive Cancer	1 (3)	-	p=1.00
Not Indicated/Unknown	0 ( - )	4 (-)	-
**High Risk HPV Screening**	***(n=6)***	***(n=3)***	
Detected	3 (50)	1 (33)	p=0.47

**Table 3:** Vaginal biopsies stratified by ethnic group (n=63).

	**African-American *(n=39)***	**Caribbean-American *(n=24)***	**Statistic**
**Position of Vaginal Biopsy**			

Upper 1/3 Vagina (w/Cx)	4 (10)	3 (12)	p= 1.0 *∗*
Vaginal Cuff (No Cx)	22 (57)	12 (50)	p= 0.62
Lower 1/3 Vagina (No Cx)	13 (33)	9 (38)	p= 0.73

**Biopsy Results (most severe site)**			

Negative/Benign	9 (23)	10 (42)	p=0.11
VaIN 1	25 (64)	8 (33)	p=0.017
VaIN 2+	5 (13)	6 (25)	p= 0.21

**Recurrent Lesions**	***(n=12)***	***(n=8)***	

No Progression	5 (42)	4 (50)	p=0.13
Regressed	0 (0)	2 (25)	p=0.15
Progressed	7 (58)	2 (25)	p=0.13

Non-progression/Regressed	5 (42)	6 (75)	
Progressed	7 (58)	2 (25)	p=0.13

**Length of Time to Last Biopsy**	***(n=12)***	***(n=8)***	

Range (years)	0.9 – 6.4	1.1 – 6.6	
Mean (years) (SD)	4.1 (1.9)	3.7 (1.8)	p=0.43

**Table 4:** Baseline characteristics, stratified by VaIN grade within ethnic group.

	**African-American**		**Statistic**	**Caribbean-American**		**Statistic**
	VaIN 1 ***(n=25)***	VaIN 2+ ***(n=5)***	Fishers Exact	VaIN 1 ***(n=8)***	VaIN 2+ ***(n=6)***	Fishers Exact
**AIDS**	20 (80)	5 (100)	p=0.37	7 (88)	6 (100)	p=0.57
**ARV**	24 (96)	5 (100)	p=0.83	8 (100)	5 (83)	p=0.42
**VL: Detectable **	***(n=21)***	***(n=5)***		***(n=8)***	***(n=6)***	
(>200 copies/mL)	4 (19)	0	p≤0.001	4 (50)	1 (17)	p=0.23
**CD4 <200**						
(cells/*μ*L)	4 (16)	0	p=0.21	2 (25)	1 (17)	p=0.46
**Cigarette Use**	15 (60)	3 (60)	p=0.37	0	1 (17)	p=0.42
**Illicit Drugs**	10 (40)	4 (80)	p=0.11	0	0	-
**Hysterectomy**	23 (92)	4 (80)	p=0.36	8 (100)	4 (67)	p=0.16
Fibroids	10 (44)	1 (25)	p=0.35	5 (63)	1 (25)	p=0.11
Dysplasia/Cancer	3 (13)	1 (25)	p=0.44	1 (13)	3 (75)	p=0.15

## Data Availability

The data used to support the findings of this study are available from the corresponding author upon request.

## References

[1] Fu S., Shi N., Wheler J. (2015). Characteristics and outcomes for patients with advanced vaginal or vulvar cancer referred to a phase I clinical trials program: the MD Anderson cancer center experience. *Gynecologic Oncology Research and Practice*.

[2] Hacker N. F., Eifel P. J., Van Der Velden J. (2012). Cancer of the vagina. *International Journal of Gynecology and Obstetrics*.

[3] Frega A., Sopracordevole F., Assorgi C. (2013). Vaginal intraepithelial neoplasia: A therapeutical dilemma. *Anticancer Reseach*.

[4] Society for Gynecologic Oncology [SGO]. Vaginal cancer: Risk factors. 2018. Available from https://www.sgo.org/patients-caregivers-survivors/caregivers/vaginal-cancer-risk-factors/

[5] Wu X., Matanoski G., Chen V. W. (2008). Descriptive epidemiology of vaginal cancer incidence and survival by race, ethnicity, and age in the United States. *Cancer*.

[6] De Vuyst H., Clifford G. M., Nascimento M. C., Madeleine M. M., Franceschi S. (2009). Prevalence and type distribution of human papillomavirus in carcinoma and intraepithelial neoplasia of the vulva, vagina and anus: A meta-analysis. *International Journal of Cancer*.

[7] Jentschke M., Hoffmeister V., Soergel P., Hillemanns P. (2016). Clinical presentation, treatment and outcome of vaginal intraepithelial neoplasia. *Archives of Gynecology and Obstetrics*.

[8] Gunderson C. C., Nugent E. K., Elfrink S. H., Gold M. A., Moore K. N. (2013). A contemporary analysis of epidemiology and management of vaginal intraepithelial neoplasia. *American Journal of Obstetrics & Gynecology*.

[9] Henson D., Tarone R. (1977). An epidemiologic study of cancer of the cervix, vagina, and vulva based on the Third National Cancer Survey in the United States. *American Journal of Obstetrics & Gynecology*.

[10] Wharton J. T., Tortolero-Luna G., Linares A. C. (1996). Vaginal intraepithelial neoplasia and vaginal cancer. *Obstetrics and Gynecology Clinics of North America*.

[11] Woodruff J. D. (1981). Carcinoma in situ of the vagina. *Clinical Obstetrics and Gynecology*.

[12] Diakomanolis E., Stefanidis K., Rodolakis A. (2002). Vaginal intraepithelial neoplasia: Report of 102 cases. *European Journal of Gynaecological Oncology*.

[13] Rome R. M., England P. G. (2000). Management of vaginal intraepithelial neoplasia: A series of 132 cases with long-term follow-up. *International Journal of Gynecological Cancer*.

[14] Sillman F. H., Fruchter R. G., Chen Y.-S., Camilien L., Sedlis A., McTigue E. (1997). Vaginal intraepithelial neoplasia: Risk factors for persistence, recurrence, and invasion and its management. *American Journal of Obstetrics & Gynecology*.

[15] Lamos C., Mihaljevic C., Aulmann S. (2016). Detection of human papillomavirus infection in patients with vaginal intraepithelial neoplasia. *PLoS ONE*.

[16] Centers for Disease Control (CDC), 2009 March 24. Guidelines for prevention and treatment of opportunistic infections in HIV-infected adults and adolescents. Recommendations from the CDC, NIH, and the HIV Medicine Association of Infectious Diseases Society of America 48(1):198. Retrieved from https://www.cdc.gov/mmwr/preview/mmwrhtml/rr58e324a1.htm 19357635

[17] Massad L. S., Xie X., Greenblatt R. M. (2012). Effect of human immunodeficiency virus infection on the prevalence and incidence of vaginal intraepithelial neoplasia. *Obstetrics & Gynecology*.

[18] Smeltzer S., Yu X., Schmeler K., Levison J. (2016). Abnormal vaginal pap test results after hysterectomy in human immunodeficiency virus-infected women. *Obstetrics & Gynecology*.

[19] CDC. HIV/AIDS Surveillance Report, 2016. Vol. 28. https://www.cdc.gov/hiv/library/reports/hiv-surveillance.html. Published November 2017

[20] Kobetz E., Menard J., Barton B., Pierre L., Diem J., Auguste P. D. (2009). Patnè en aksyon: Addressing cancer disparities in Little Haiti through research and social action. *American Journal of Public Health*.

[21] Menard J., Kobetz E., Maldonado J. C., Barton B., Blanco J., Diem J. (2010). Barriers to cervical cancer screening among Haitian immigrant women in Little Haiti, Miami. *Journal of Cancer Education*.

[22] Baeker Bispo J. A., Seay J. S., Kobetz E. K. (2017). The Use of Commercial and Plant Products in the Vaginal Hygiene Practices of Haitian Women: A Latent Class Analysis. *Annals of Epidemiology*.

[23] Seay J. S., Mandigo M., Kish J., Menard J., Marsh S., Kobetz E. (2017). Intravaginal practices are associated with greater odds of high-risk HPV infection in Haitian women. *Ethnicity & Health*.

[24] Corporation IBM. *IBM SPSS Statistics*.

[25] Rasmussen C. L., Sand F. L., Hoffmann Frederiksen M., Kaae Andersen K., Kjær S. K. (2018). Does HPV status influence survival after vulvar cancer?. *International Journal of Cancer*.

[26] Smith J. S., Backes D. M., Hoots B. E., Kurman R. J., Pimenta J. M. (2009). Human papillomavirus type-distribution in vulvar and vaginal cancers and their associated precursors. *Obstetrics & Gynecology*.

[27] Buchanan T. R., Graybill W. S., Pierce J. Y. (2016). Morbidity and mortality of vulvar and vaginal cancers: Impact of 2-, 4-, and 9-valent HPV vaccines. *Human Vaccines & Immunotherapeutics*.

[28] Society for Gynecologic Oncology [SGO]. The crisis in gynecologic cancer clinical trial access. 2017. Retrieved from https://www.sgo.org/wp-content/uploads/2012/09/SGO-Clinical-Trial-Crisis-FINAL.pdf

